# Data on the knowledge, attitude, and performance of Ph.D. students attending an educational course (Tehran, Iran)

**DOI:** 10.1016/j.dib.2018.08.081

**Published:** 2018-08-30

**Authors:** Zohreh Sohrabi, Hamid reza Koohestani, Seyedeh Zahra Nahardani, Mohammad Hasan Keshavarzi

**Affiliations:** a Department of Medical Education, Faculty of Medicine, Vice-Director, Center for Educational Research in Medical Sciences (CERMS), Iran University of Medical Sciences (IUMS), Tehran, Iran; b Department of Medical Education, Faculty of Medicine, CERMS (Center of Educational Research in Medical Sciences), Iran University of Medical Sciences, Tehran, Iran; c Department of Medical Education, Saveh University of Medical Sciences, Saveh, Iran

**Keywords:** Instructional design, Basic Medical Sciences students, Kirkpatrick׳s model, Knowledge

## Abstract

The Department of Medical Education of Iran University of Medical Sciences organized a workshop on empowerment and familiarity with the teaching and learning principles. The data presented here is based on the effectiveness of this workshop. This data was acquired from 29 postgraduates who take part in a two-day educational course and instructional design workshop. The samples were selected by convenience sampling. Data collection tool was a questionnaire that consisted of 5 questions for demographic variables, 20 question about attitude and satisfaction, 30 questions on knowledge as pretest and posttest, and 3 questions about behavior and performance. The descriptive statistics of data were analyzed using SPSS-14. The mean score of pre-test and post-test in case of knowledge, attitude, and performance in teaching and instructional design were calculated. In addition, the viewpoints of educational departments on the third level of Kirkpatrick׳s model i.e. the students’ post-workshop behavior change (transferring learning to the workplace) were obtained.

**Specifications Table**

**Table:** 

Subject area	Health education
More specific subject area	Medical education
Type of data	Table and Figure
How data was acquired	The data in this article obtained from 29 postgraduates who took part in a two-day educational course and instructional design workshop. The participants filled a questionnaire based on Kirkpatrick׳s Model.
Data format	Raw and analyzed
Experimental factors	Content validity was used for validating the tool, and split-half method and Cronbach׳s alpha (r = 0.88) were used for measuring the reliability.
Experimental features	Kirkpatrick׳s Model consisted of 5 questions for demographic variables, 20 question about attitude and satisfaction, 30 questions on knowledge as pretest and posttest, and 3 questions about behavior and performance. The questions had a five-point Likert scale.
Data source location	Tehran, Tehran province, Iran
Data accessibility	Data are included in this article

**Value of the data**

• Evaluating the effectiveness of educational courses not only enables managers to have a clearer image of the quantity and quality of educational programs, but it can also help the organization׳s policymakers and educational staff to have better awareness of the positive and negative aspects of a program [1], [2], [3].
• This data could be useful to improve the knowledge, attitude and performance of students during educational courses.
• This data can be used by managers of educational organizations to conduct more effective courses for students.

## 1 Data

From 29 participants, 18 of them were females (61.9%) and 11 were males. The participants’ age ranged from 28 to 40 (the mean age was 31.35). They were from different departments: parasitology (3 participants), genetics (2 participants), immunology (3 participants), biochemistry (3 participants), pharmacology (2 participants), physiology (5 participants), microbiology (3 participants), anatomy (5 participants), and medical physics (3 participants).

**Fig. 1:**
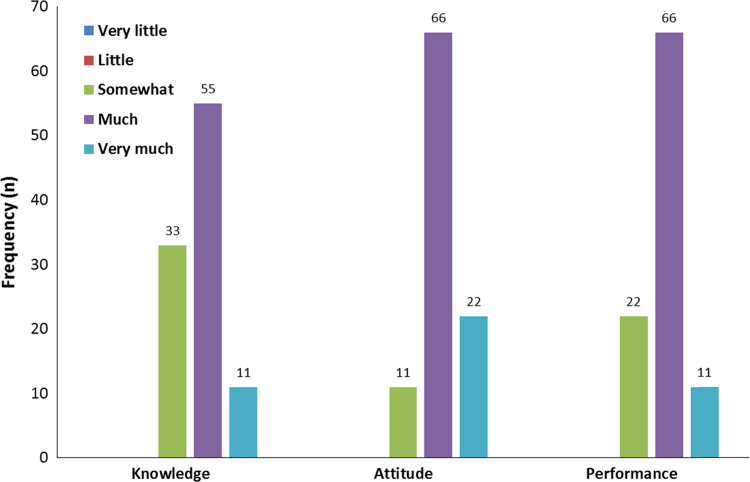
The frequency of the viewpoints of educational departments on students’ post-workshop behavior change (transferring learning to the workplace).

The students’ mean scores of pre-test and post-test are provided for attitude toward teaching and educational design and learning in Table 1. As can be seen in Fig. 1, most of the educational departments believed that the students’ educational knowledge, attitude, and performance changed after holding the workshop.

**Table 1:** Mean and standard deviation of students’ pretest and posttest scores for attitude and learning.

**Variable**	**Pretest**		**Posttest**		**P-value**
	**Mean**	**Standard deviation**	**Mean**	**Standard deviation**	
Attitude (Kirkpatrick׳s first level)	29	2.6	36	3.77	0.01
Learning (Kirkpatrick׳s second level)	14	0.86	23	0.24	0.001

## 2 Experimental design, materials and methods

The data was acquired from a Quasi-experiment with a pre-test/post-test design conducted in the academic year of 2017–2018 in the Medical School of Iran University of Medical Sciences. The statistical population included basic sciences Ph.D. students of Iran University of Medical Sciences. The inclusion criterion was the willingness for participation. The exclusion criterion was the student׳s unwillingness to continue. Twenty-nine students were selected by convenience sampling. The data collection tool was a five-section researcher-made questionnaire. The first part deals with individual and demographic characteristics (such as age, gender, academic semester, major, and history of attending a workshop). The second part includes 10 questions about satisfaction with the workshop (Kirkpatrick׳s first level) on a five-point Likert scale (ranging from very little to very much). The third part includes 10 questions about attitude towards educational design and teaching methods (Kirkpatrick׳s first level) with a five-point Likert scale (ranging from completely agree to completely disagree). The fourth part includes 30 multiple-choice questions about evaluating the learning (Kirkpatrick׳s second level), educational design, and teaching methods. Part five includes 3 questions on behavior change (Kirkpatrick׳s third level) with a five-point Likert scale (ranging from very much to very little). The scores of 1–5 have been defined for different levels of the questionnaire in Likert scales. The minimum and maximum scores of satisfaction questionnaire were 10 and 50, respectively. The classification was defined as such: 10–18 very dissatisfied; 18–26 dissatisfied; 26–34 neither satisfied nor dissatisfied; 34–42 satisfied; and 42–50 very satisfied. Content validity was used for validating the questionnaire, and split-half method and Cronbach׳s alpha (r = 0.88) were used for measuring the reliability.

Before the workshop, the participants’ attitude and knowledge were measures by using attitude and learning tool. At the end of the workshop, the students’ attitude (satisfaction) and learning about educational design and teaching method as well as their satisfaction with the workshop were measured once more.

The data were analyzed in SPSS by using descriptive statistics (mean and standard deviation). For preserving the ethical considerations, the participants were justified about the purpose of the data acquisition. Moreover, they were assured that the information would remain confidential and there was no need for writing their names. This project was approved in the ethics committee of Iran University of Medical Sciences.
